# Increasing the use of biological pesticides in integrated pest management programs

**DOI:** 10.3389/finsc.2025.1552361

**Published:** 2025-10-23

**Authors:** Pamela G. Marrone

**Affiliations:** Invasive Species Corporation, Davis, CA, United States

**Keywords:** biologicals, biopesticides, bioprotection, biocontrol, natural products, microbials

## Abstract

While biopesticides have been around for 70 years, starting with *Bacillus thuringiensis* bioinsecticides, they are experiencing rapid growth as the products have gotten better and more science-based and there are more restrictions on synthetic chemical pesticides. The growth of biopesticides is projected to continue to outpace that of chemical pesticides, with compounded annual growth rates of 10%–20% versus single digits for chemicals. When integrated into pest management programs, biopesticides offer the potential for higher crop yields and quality than chemical-only programs. Added benefits include the reduction or elimination of chemical residues, therefore easing export, delay in the development of resistance by pests and pathogens to chemicals, shorter field re-entry, biodegradability and lower carbon footprint and greenhouse gas emissions, and low risk to non-target organisms, including pollinators. However, many challenges still exist to drive further the adoption of biopesticides. These include lack of awareness and education in how to test and deploy their unique modes of action in integrated programs, resulting in lingering perceptions about performance and cost-effectiveness. This article addresses these issues with suggestions on how to increase farmer and key influencer confidence in the deployment of biologicals in integrated pest management (IPM) programs, including adjusting IPM thresholds and practice based on the modes of action of biopesticides compared to synthetic chemicals.

## 1 Introduction

It has been several decades since the concept and definition of IPM were formed. Despite the time that has passed and the high growth of biopesticides globally, there are many agricultural cropping systems that still rely heavily on synthetic chemical pesticides, including calendar sprays rather than true IPM, where synthetic chemicals are the last resort. Not only is there a robust ecosystem of venture capital-backed startup biopesticide companies, but there are also several medium companies with biopesticide revenues exceeding US $100 million and large agrichemical companies with more than US $500 million in biopesticide revenues. However, farmer surveys in both row crops and high value specialty crops show that approximately half of all farmers do not use biopesticides because they lack awareness of the category or do not understand how to use them. Key influencers such as crop consultants, pest control advisors, university extension specialists, and farm advisors may not also fully understand the category and may be testing these unique products with trial protocols designed for chemical pesticides. As such, they receive negative reviews. Biopesticides are often listed in university IPM recommendations for use only in organic farming, yet more than 70% of their use is in conventional farming. Testing biopesticides and integrating them into IPM programs based on their unique modes of action will help increase the confidence in their use to the benefit of IPM programs, growers’ return on investment, human and nontarget safety, and the environment.

## 2 Biopesticide definitions and categories

It is important to define biopesticides as there is often confusion as to the types of products regulated as biopesticides versus other natural or nature-derived materials.

### 2.1 Microbial biopesticides

Microbial biopesticides contain microorganisms (bacteria, insect viruses, fungi, actinomycetes) and protozoa that function as biocontrol agents, affecting the pest directly or indirectly through the compounds that they produce. The most well-known and largest microbial biopesticide is strains of *Bacillus thuringiensis* (Bt) for caterpillar, mosquito, and beetle control, having been commercialized more than 60 years ago and still the most prominent product group on the market because Bt products are low cost and used for resistance management in fruits and vegetables. Microbials for plant disease management were extensively reviewed in a new book (1). Note that entomopathogenic nematodes, while used in insect biocontrol, are exempt from regulation by the EPA as biopesticides and as such are not included in this category.

Non-living microorganisms have been regulated as microbials in the past. Examples include Marrone Bio Innovations’ biomolluscicide (nonviable *Pseudomonas fluorescens* CL45A) for invasive zebra and quagga mussel control in water and pipes. Other examples include bioinsecticides based on new species of bacteria, heat-killed *Burkholderia rinojensis* A396 (2) and Valent Bioscience’s nematicide from the killed fungus *Myrothecium verrucaria*. Today, the EPA’s BPPD may classify some of these types of biopesticides as biochemicals (see below) if the metabolites are excreted versus cell-borne and the primary cause of the bioactivity.

### 2.2 Biochemical biopesticides

Biochemicals contain naturally occurring substances that control pests, weeds, and plant pathogens. These natural substances include potassium bicarbonate, fatty acids, some plant extracts (including some essential oils), and pheromones for insect mating disruption. Not all natural substances are regulated as biochemicals. Just because something is natural does not mean it is low risk. Therefore, the BPPD has a Biochemical Classification Committee to determine if a natural substance is a biopesticide or should be registered as a chemical pesticide in the RD. To be declared a biochemical biopesticide, the active ingredient must have a nontoxic mode of action to the target pest or pathogen. This can be confusing. Examples of nontoxic modes of action include induced systemic resistance and systemic acquired resistance for control of plant pathogens (e.g., knotweed extract, seaweed extracts), suffocation and desiccation (e.g., diatomaceous earth and mineral and essential oils), growth regulators (e.g., azadiractin and neem-based products), and mating disruption pheromones.

### 2.3 Emerging technologies

The EPA recently formed an Emerging Technologies branch (ET) to handle new technologies. These include plant-incorporated protectants (PIPs) (genetically engineered crops for pest control), which have been registered in the BPPD for many years and are not considered biopesticides, RNAi-based products, novel peptides, and genetically engineered microorganisms. The EPA registered the first RNAi product as a biopesticide, produced by Greenlight Biosciences, for Colorado potato beetle. There are several other companies working on RNAi products such as RNAissance Ag, RNAWay, Innatrix, Pebble Labs, Agrospheres, Trillium Ag., and Micropep received a designation for their novel peptide as “biochemical-like.” Some companies are engineering RNAi genes for the control of plant pathogens into plant-colonizing organisms for delivery onto the foliage or roots (e.g., Robigo).

### 2.4 What are not biopesticides

Substances that are natural with a toxic mode of action are regulated as chemicals in the Registration Division (RD). Examples include the microbial metabolites spinosyns and avermectin (produced in fermentation) and pyrethrins (extracted from a type of *Chrysanthemum* plant), which all have toxic modes of action because they work specifically on the insect’s nervous system with some cross-over to mammalian systems. Another example is D-limonene from citrus, which is not registered as a biochemical biopesticide but instead as a chemical through the RD. However, farmers may perceive products like spinosyns as biopesticides because they are natural products and have formulations that are listed for organic farming (e.g., Pyganic^®^ and Entrust^®^). Considering this rigorous classification, biopesticides are considered the least toxic and lowest-risk pesticide category of all pesticides. More detail on the regulatory process can be found in (12, 13).

## 3 Market size and growth

The market for biopesticides (also called biocontrol or bioprotection) is growing at a compounded annual growth rate (CAGR) of approximately 12% globally (3). Global market value is approaching US $9.0 billion, compared to global chemical pesticide market (crop and non-crop) of US $78 billion with growth rates in the single digits (4) (https://www.spglobal.com/commodityinsights/en/ci/research-analysis/global-crop-protection-market-provisionally-up-6-in-2022.html). According to the 2024 CropLife 100 survey, 63% of the nation’s top agricultural retailers saw sale increases between 1% and more than 5% for their biological offerings for grower-customers, beating out the growth of all other (chemical) categories (5). Given this growth, the math indicates that biologicals will reach the size of the chemical pesticide market by 2040 (6).

According to Trimmer (3), there are multiple companies with revenues approaching US $500 million (e.g., Bayer, Syngenta, UPL) and more than 10 companies, mostly pure play biologicals companies, that now have >US $100 million revenue (e.g., FMC, Koppert, BioFirst Group, Rovensa Next, Certis Biologicals, Bioceres Crop Solutions, Valent Biosciences). While North America is currently the largest region, Latin America is rapidly gaining market and will take the lead by the end of the decade. Brazil has become the first market where biopesticides have been widely adopted in row crops including corn, soybean, sugarcane and cotton. Extensive adoption in soybeans—combined with the continued expansion of soybean-planted hectares—is a key driving force in this crop. Biological nematicides are currently the leading choice of Brazilian producers for controlling these parasites, accounting for 75% of the total market, according to Agropages (7) who cited data from the FarmTrak study by Kynetec. The survey revealed that investments in this type of nematode control have already reached a value of BRL1.2 billion (approximately US $245 million). Chemical solutions were BRL395 million (USD $80 million). For comparison, in 2015, chemicals dominated the nematode control market with a 94% share, while biologicals had only 6%. The growth in Brazil can be attributed to a rapid regulatory process, averaging 13 months (8), government support that provides economic incentives, basic research, grower education, and an improved regulatory process to facilitate biological adoption. Today, Latin America is the fastest-growing biologicals market in the world, led by this rapid expansion in Brazil.

Many of the products are used as seed treatments to enhance control and yield. Grandview Research and other market research companies estimate the biological seed treatment at USD $1.6 billion in 2024 with 12.6% CAGR (9). Seed treatments in the United States are used on hundreds of millions of acres of corn, cotton, and soybeans, typically stacked with chemical treatments on the seed to enhance control and yield. Biopesticides are widely used in the US and Western Europe fruit and vegetable markets. The early success and expansion in these crops were the engine that fueled the early growth of the global biocontrol market. These markets are now maturing, and growth is slowing. Europe could be a large growth market since hundreds of chemical pesticides are restricted or removed from the market, but the regulatory process for biopesticides is designed for chemical pesticides and takes several years and millions of dollars. The US market has slowed as the once speedy regulatory process has slowed down due to lack of staff at the Environmental Protection Agency’s (EPA) Biopesticide Pollution Prevention Division (BPPD) to handle the explosion of new active ingredient (AI) submissions. To compare, the BPPD has approximately 80 new AIs compared to approximately 10 for the chemical division (RD) (10).

In 2024, the media company The Mixing Bowl mapped the number of biopesticide companies (https://www.mixingbowlhub.com/landscape/2024-crop-biocontrol-landscape). The number and diversity of companies around the globe is astonishing, and it is unlikely that most IPM practitioners are aware of the companies, their products, and the many innovations coming to market in the next 1 to 5 years. Attention to what is happening in biopesticides can provide IPM practitioners new solutions to better solve growers’ problems than chemicals alone, given the tiny number of new chemical AIs relative to the number of new biopesticides.

## 4 Factors driving biopesticide growth

There are several factors driving the growth of biopesticides. These drivers were extensively reviewed in Marrone (11), including (a) better grower return on investment (ROI) in integrated programs compared to chemical-only programs, (b) application of new scientific tools and discovery of new technologies, (c) resistance and residue management, (d) reduced carbon footprint and greenhouse gas emissions, (e) enhanced soil health, (f) labor flexibility (short re-entry intervals), (g) growth of organic farming, and (h) use of precision technology to aid in biopesticide delivery and IPM.

The advent of artificial intelligence and machine learning will drive (b) above. Once a microorganism is isolated, the question arises as to how scientists select which ones to test against the target pest. In the past, it was based on microscopy and the scientists’ knowledge of which colonies to select from a petri plate. Now, 16S RNA sequence or a full gene sequence can be determined for each microorganism, enabling scientists to select ones to test based on the 16S RNA results. After obtaining the 16S RNA or full genome sequence, new tools guided by artificial intelligence (AI) and machine learning (ML) can be applied to mine data known about that sequence and taxonomy. Information can quickly be obtained, including possible metabolites produced by the microorganism, known activity against pests and pathogens, toxicity/pathogenicity to mammals and other organisms, growth-promoting effects on plants, nitrogen fixation, and carbon sequestration. This information then allows the scientists to make an informed decision to select microorganisms for maximum biodiversity that are non-toxic, non-pathogenic, and most likely to be active against the target pest. For new species, these may be unknown, but relatedness to known species and strains can provide clues. This is the approach we have taken at the Invasive Species Corporation. In less than 9 months, we have found microorganisms with activity on burrowing shrimp (for a Washington State Department of Agriculture project to find biological solutions to control shrimp damaging oyster farms), algae, and weeds. Our first environmental sample isolations resulted in 23 different microbial orders. The “hit rate” (microbes with activity against a target) is much higher with this information-driven approach. At our previous company, Marrone Bio innovations, when AI/ML-driven data mining tools were not available, our “hit rate” for herbicides was 1.54% and for algaecides 0.67% (12), while at ISC it is 30.9% for herbicides, 16.67% for algaecides, and 38% for shrimp. Genomics and machine learning can also be applied to microbial fermentation process optimization and formulation to more quickly improve the fermentation for yield and metabolite optimization and for finding the best formulation to stabilize the microorganism to plant extract or other natural substance.

The barriers to further growth have been reviewed in Marrone (13), which are updated and discussed below.

## 5 Understanding the development cycle of biopesticides

The difference between the development of a biopesticide versus a chemical pesticide was reviewed in Marrone (11–13). However, it is worth repeating here because this is one of the biggest misunderstandings of biopesticides and can lead to lack of confidence in the product category. When a chemical pesticide is launched, it has $300 million and 12 years behind it (24). Because of the shorter time to develop and launch a biopesticide based on the streamlined regulatory process, a biopesticide may be launched in approximately 4 to 5 years. As such, it will not have thousands of field trials like a chemical. There may be hundreds of field trials and only US data with a narrow commercial label with a small number of crops and pest uses compared to global trials supporting a broad finetuned label for a chemical. Some key influencers have criticized the lack of data compared to a chemical pesticide and therefore will not recommend the biopesticides to growers (15). This results in the company, usually a small venture capital-backed startup, doing their own trial demonstrations with growers to gain sales revenues needed to support the next round of financing.

What this author has found in pioneering biopesticides since starting three previous companies in 1990, 1995, and 2006 is that there are early adopter growers eager to try the biopesticide against pests or pathogens lacking in effective solutions often due to resistance, chemical restrictions, or simply a lack of products—for example, when we launched Serenade^®^ biofungicide in 1998 at AgraQuest, a Florida grower put it on all of his tomato acreage in combination with copper because he needed a better solution. When combined with copper, the grower had better control than either alone. Abbasi and Weselowski (16) found that Serenade in tank mixed with copper hydroxide reduced disease severity on foliage of 4 years and increased the total fruit number in 2 years. Another example is a table grape grower in California with a late infection of *Botrytis* bunch rot and who was ready to ship to Europe. At that time, no chemical pesticides were approved for pre-harvest applications; he saw that Serenade was just made available and saved his crop and exported on time as there are no pre-harvest restrictions with most biopesticides. Another example is when we launched Venerate^®^ at Marrone Bio Innovations in 2014; to our surprise, a peach grower in Georgia applied it for stink bugs because the crop was close to harvest and chemicals could not be used for this late infestation. In all three cases (and there are many other examples), the growers were happy with the performance, resulting in significant economic gain and crop loss protection. Therefore, it would behoove those practitioners who advise and recommend pest management solutions to growers to understand and have knowledge of the plethora of biopesticide products that are registered and could be used to enhance IPM programs rather than waiting for several more years of stand-alone data.

Another key point for biopesticides, particularly with microbials, is that they can be continually improved through manufacturing and formulation improvements. This results in a new and improved version every 3 to 4 years or so—for example, the first version of Grandevo^®^ bioinsecticide (12) was a wettable powder; the present formulation is a water-dispersible granule packed with a higher level of the key pesticidal metabolites. Having the full genome sequence of a microorganism allows understanding of the microbial physiology and genes associated with pesticidal metabolite production, resulting in increased yields of not just cells but also the pesticidal compounds. An example is the microbial bioinsecticide from the new species *Burkholderia rinojensis* (2) where the pesticidal compounds were increased 150-fold, resulting in lower use rates and higher efficacy (12), which won the President Green Chemistry Award for the seed treatment product called RinoTec™ (https://www.epa.gov/greenchemistry/2024-green-chemistry-challenge-winners). Therefore, IPM practitioners should not dismiss a product they tested 5 years ago as ineffective and should be aware of the ongoing improvements to biopesticide products and give them another look.

## 6 Grower surveys about biologicals

The results of recent surveys are consistent with past surveys (11, 13). Stratovation Group (17) conducted surveys of row crop, which was updated in 2024. While just under half (45%) of all row crop producers say that they currently purchase or use agricultural biological products on their fields, that number is up to 8% from 2022’s benchmark survey. Farmers who use biologicals on their row crop fields continue to rate them positively (7.4/10 in the 2022 study), and increased yield was by far the most common metric of success among biological users (85%), followed by profitability at (45%). Among biologicals users, cost was the biggest negative for using biologicals. Of 270 non-users, producers who have never used biologicals believe that they are not proven or have a lack of knowledge on the subject. Of high-value specialty growers from a Stratovation survey in 2023 of biologicals users, the growers rated them highly, 7.14/10 (18). Like row crop growers, yield was ranked as the top benefit and cost was what users liked the least. Of the non-users, specialty crop growers lack knowledge or think that they are not proven. Furthermore, 53% of the growers surveyed said no to the question, “Have you been educated about biologicals? Such as the benefits, limitations, or available options?” This certainly presents an opportunity to increase the use of biologicals by increasing the knowledge through education of key influencers who make recommendations to farmers and to farmers themselves.

Baker et al. (19) elucidated the challenges of the adoption of IPM and biocontrol. Their conclusions are consistent with these grower surveys: “Longstanding obstacles to IPM adoption in general are also barriers to biological control use, including direct costs outweighing direct benefits to users; poor recognition and accountability for indirect costs of tactics with greater risks to health and environment; lack of incentives to overcome high direct costs to users despite indirect benefits to the public; incomplete information; complexity…” They state that “strategies to speed adoption include increased education and extension on proven, ready-to-use biological control options; full cost and benefit accounting for biologically based alternatives to chemical controls; and public and private sector policies to encourage biological control and reduce reliance on chemical controls.”

## 7 Testing and using biopesticides based on their modes of action

### 7.1 Adjusting IPM systems to best use biopesticides

Biopesticides are often not used appropriately based on their unique modes of action. The established rules on IPM often do not apply. One such rule is to allow pests to build up to economic threshold or economic injury level (EIL) before treating. Biopesticides such as Marrone Bio’s Grandevo^®^ based on the new species of bacterium *Chromobacterium subtsugae* (20) are not highly effective when used to knockdown pests at an EIL. *Chromobacterium subtsugae* rapidly stops feeding and inhibits reproduction and should be applied early in the season before the pests build up. There are many biofungicides on the market today, including several *Bacillus*-based products that prevent pathogen spore germination and/or trigger systemic acquired resistance or induced systemic resistance. They are not curative and will not perform well when applied after the infection is well along. Therefore, it is critical to educate the manufacturers’ sales teams, end-users, and key influencers on using the product early before pests and populations are present or populations increase. If pest populations are already high, start with another insecticide with contact activity with more knockdown effects, followed by the product with effects on feeding, pest development, and fertility. These novel biopesticides can be very successful in conventional resistance and residue management programs and can enhance the overall program in plant health and yields.

### 7.2 Adjusting testing protocols

The single most significant barrier to the adoption and growth of biopesticides is that they are often not tested based on understanding their modes of action but instead tested like curative or fast-acting chemical pesticides. Biopesticides are usually tested standalone with comparisons to the best mixtures of chemical pesticides using evaluation criteria for chemical pesticides, such as area under the disease progress curve, number of leaf spots, or number of pests. These criteria in stand-alone trials certainly should be used to assess biopesticides as well, but they are not the sole measures that should be used for the evaluation of biopesticides. Biopesticides are best used in rotations and tank mixtures or early in the season before pest or pathogen buildup and before harvest to manage residues. Integrated programs can increase growers’ bottom lines with better yields and quality, which is what is driving the growth and adoption of biopesticides by conventional growers. Therefore, it is critical to assess data on plant damage, not just pest or leaf spot counts, and yield and quality should be incorporated into testing regimens since marketed yield and quality are what growers care about—for example, Grandevo^®^ bioinsecticide stops feeding in less than 1 min, and reproduction is reduced (21), but pest mortality is slow—typically in 7 to 10 days. A Cornell study with Venerate^®^ bioinsecticide showed complete protection of apples from stinkbugs without much immediate adult mortality (22). Attention should also be paid to the water volume used as too much water will dilute the dosage needed to provide an effective dosage of the biopesticide. Some commonly used adjuvants can reduce the efficacy of biopesticides (21); therefore, education about the particular requirements for efficacious use is critical.

Since growers rarely use any pesticide standalone but instead mix and match to get best performance and to manage resistance, once some activity is seen with a biopesticide in standalone trials, trials should be conducted in integrated programs with chemicals in tank mixtures or rotations. An objection to touting the benefits of biopesticides in integrated programs is that the chemical could be doing the heavy work in the program. How this matters is through the added benefits of resistance and residue management, shorter worker re-entry, and zero-day pre-harvest intervals that can make a compelling value proposition to growers (23). Other benefits include enhanced soil health and biodiversity, for example, recording any increases in beneficial insects and mites and changes in biodiversity and micro functionality of the soil microbiome before and after treatment. Buyers and governmental agencies are increasingly asking what grower practices do to carbon footprint, greenhouse gas emissions, and nutrient density. For these factors, biopesticides have advantages and can be and are being measured compared to chemical pesticides, with reductions of greenhouse gas emissions of 80%–90% compared to chemical pesticides (13).

### 7.3 Using 0.05 for testing significance

Given the variability of natural systems when testing any crop protection product, is the standard 0.05 *p*-value to determine significance when conducting biopesticide trials the appropriate measure? An article recently suggested that it is not (https://www.usatoday.com/story/special/contributor-content/2024/09/13/cxc-exposes-the-hidden-dangers-stifling-innovation-the-overreliance-on-statistical-significance/75207306007/). Dr. François Lamoureux, CEO of CXC™, specializes in advancing promising technologies to a total readiness level (TRL) of 7.5 or 8 out of 9. The company sponsors university research, providing the resources needed to further develop groundbreaking ideas. However, after years of involvement in this space, François and his team discovered a troubling disconnect between the pressures of academia and the realities of a for-profit market economy. The *p*-value is meant to indicate whether the observed data is compatible with a given statistical model, typically under the assumption that the null hypothesis is true. While this might sound reasonable, the stringent application of this threshold can lead to the dismissal of potentially transformative research simply because it does not meet an arbitrary cutoff. “The author concludes: “This shift in perspective is vital for the future of innovation. While *p*-values and statistical significance have their place, they should not be the sole determinants of a project’s worth. Instead, a more holistic approach that considers economic significance, effect size, and empirical impact is needed to ensure that promising innovations are not prematurely discarded.”

The author of this paper has overseen many thousands of biopesticide field trials. Many reports from researchers conclude that the biopesticide did not separate from the untreated (using 0.05 *p*-value). However, quite frequently the widely used chemical pesticide did not separate either! This can be particularly problematic for trials measuring marketable yield; when the data are redone using a 1.0 *p*-value, trends that would be dismissed using *p* = 0.05 can often be seen.

### 7.4 A model: Cornell University plant pathology, Katie Gold lab

Dr. Katie Gold’s program has done an exemplary job of providing the basic educational information about biopesticides, specifically biofungicides for grape disease management (14; https://cals.cornell.edu/news/2022/05/grapes-101-biopesticides). The information speaks to the rationale for using biopesticides and how to use them based on their modes of action. The basic educational information is followed by standalone field trial data for grape powdery and downy mildew control, comparing several biofungicides to chemical standards. Most of the time, the biofungicides were not quite as good as the chemical standards in percent control, but the integrated programs of the biologicals and chemical fungicides performed well. The conclusions: “In both these cases, we found that using a biopesticide in rotation reduced overall conventional chemistry usage by half while maintaining highly effective disease control! Integrating biopesticides into a disease control program reduces the control pressure placed on conventional chemistries, slowing the development of fungicide resistance in target pathogen populations. Protecting the longevity of highly effective, conventional chemistries is essential for the long-term health and sustainability of the New York grape industry. Using biopesticides in your early or late season disease control program will help ensure that the traditional chemistries we rely on for robust powdery mildew and downy mildew control during the critical period of pre- to post-bloom will be in our toolbox for years to come.”

### 7.5 Western Growers’ biological testing initiative

California is unique in the United States in that it restricts or bans more chemical pesticides than any other state—for example, it was the first state to restrict chlorpyrifos, has severe restrictions on soil fumigants, and is working on neonicotinoid restrictions. In 2023, the California Department of Pesticide Regulation and California Department of Food and Agriculture published a sustainable pest management (SPM) roadmap (https://www.cdpr.ca.gov/docs/sustainable_pest_management_roadmap/). In this roadmap, the “north star” is “by 2050, California has eliminated the use of priority pesticides by transitioning to sustainable pest management practices.” Roadmap recommendations include educating the key influencers—university extension personnel, pest control advisors and crop consultants—on the principles of SPM and accelerating the approval of low-risk products, particularly biopesticides. As such, California growers, facing even more chemical restrictions, clamored for ways to accelerate the adoption of registered biopesticides. With that, Western Growers, a California-based grower trade group, initiated a program to test biopesticides in more realistic on-farm situations (https://www.linkedin.com/posts/pam-marrone-110ab6_biopesticide-activity-6901940317654806528-g9bA/). The results of these trials would then provide growers with more confidence to try the biopesticide solutions. Growers were surveyed to understand their worst pests and disease problems. Despite using chemical pesticides, a long list of grower needs was assembled. These included control of resistant weeds and weeds in organic production, glassy winged sharpshooter, large Hemiptera such as leaf-footed bugs, brown marmorated stink bug, and green stink bug; thrips, plant parasitic nematodes, soil pests/replant disorder (tree crops), bacterial diseases such as bacterial blast (*Pseudomonas syringae*) for tree crops and *Xanthomonas* of tomato, pepper, and walnuts; fungal canker diseases of tree and vine crops, Oomycetes such as *Phytophthora* and downy mildew of leafy greens; and *Fusarium* of cotton, tomato, lettuce, and other vegetables. Products listed as needing alternatives included chlorpyrifos and other organophosphates, neonicotinoids, chemical fumigants (pre- and post-harvest), pyrethroids, sulfur, copper and antibiotics.

Driscoll’s Berries and The Wonderful Company agreed to participate in the pilot for solutions to *Lygus* bug in berries and insecticide-resistant thrips on citrus. A steering committee selected products from Lallemand, Profarm Group, Terramera, and Anatis Bioprotection. These two grower–packers were pleased with the results (unpublished). In 2023, Western Growers teamed up with a New Zealand company to expand the testing to the northern and southern hemispheres. Another RFP was published, and companies were selected for this testing (https://www.platform10.ag/news/the-2024-biologicals-cohort-announced-for-platform10/). The companies selected were AgroSpheres, Bayer, Boost Biomes, Impello Bio, Lallemand, ProFarm Group, Summit Agro, and Vestaron, not just small startups but also some established companies. Companies such as Bayer, with Serenade^®^ biofungicide, indicated that they also experience issues with how the product is tested for best efficacy (Denise Manker, personal communication).

### 7.6 The Almond Board of California biopesticide testing program

Dovetailing the Western Growers program described above, The Almond Board of California (ABC) announced a RFP (https://www.almonds.com/almond-industry/industry-news/seeking-biopesticides-evaluate-california-almonds) for companies to participate in their 2024 biopesticide testing program to address their growers’ significant disease and pest issues that arose due to chemical pesticide resistance and introduction of new pathogens. In 2024, the program kicked off with a biofungicide testing program. Experts, including this paper’s author, were to select the products for testing and to design protocols suited for their particular modes of action. Trials were to be conducted with growers on-farm and in larger blocks instead of trials commonly conducted with a few branches per tree. Then, the ABC selected two contract research organizations (CROs) to conduct the on-farm trials.

Four California-registered biopesticides selected for use in conventional almond production were elevated for efficacy against common pathogens during the spring and summer of 2024. The biopesticides evaluated were Stargus^®^ (*Bacillus nakamurai*), BotryStop^®^ WP (*Ulocladium oudemansii strain* U3), EcoSwing (extract of *Swinglea glutinosa*), and Serenade (*Bacillus subtilis* (*amyloliquefaciens* QST 713 (BM 02). Efficacy was tested on brown rot (*Monilinia laxa*), jacket rot (*Botrytis cinerea*), hull rot (*Rhizopus*, *Monilinia*, *Aspergillus*, and *Phomopsis*), alternaria (*Alternaria alternata*), and rust (*Tranzschelia discolor*). Note that Serenade was first launched in 1998 and has had several new formulations and process improvements since that time. Now marketed by Bayer Crop Science, it is not used much in conventional almonds as it is not recommended in the University of California IPM Guidelines, except for organic production. Serenade and EcoSwing performed the best of the four products and, depending on the pathogen, were approaching the performance of, equal to, or better than the reference chemical fungicides. These two products were recommended for repeat testing in 2025, not just stand-alone but with the conventional fungicides in integrated programs.

## 8 Recommendations

Baker et al. (19) listed several recommendations that are still relevant 5 years later and that can serve as a basis for some updated recommendations. The California SPM Roadmap can be applied US-wide and tailored to each state’s or region’s specific pest and disease problems. Why the necessity? Any survey of growers will tell us that despite the use of chemicals, they still have many pest and disease problems. Consumers and consumer product companies demand more sustainable pest management and crop production practices and systems that include biodiversity, soil health, and carbon footprint. Given the growth of biopesticides, their broad benefits, and the lack of new chemical AIs, there needs to be a change of paradigm on how to better integrate biologicals into IPM programs. Ultimately, the best IPM is SPM, focusing on holistic systems and ecological programs rather than reliance on pesticides, biologicals, or chemicals. However, on a continuum to this ultimate state, we can better transition to more biologicals to increase grower and key influencer confidence in these valuable tools.

Some suggestions for bold change are as follows:

A. The land grant universities should undertake a countrywide initiative to make biopesticides a more prominent choice for crop protection in their pest management recommendations (see the work of Gold and Combs (14) on biopesticides for disease management as a model).

- Work with industry trade groups such as the Biological Products Industry Alliance (www.bpia.org) and grower/commodity groups to develop educational modules on biologicals—the categories, how they are registered, current landscape of companies and products, how to test them based on their modes of action, and incorporate them into integrated programs.
- Incorporate updated testing paradigms and protocols into graduate student training and thesis work.
- Increase funding for university biopesticide testing (since small biopesticide companies do not have the budget of large multinational companies to pay researchers for trial work).
- Significantly invest in SPM-focused research and outreach so that all pest management practitioners have equal and adequate access to the support and resources necessary to develop and implement their own SPM systems.
- Provide incentives to extension personnel to integrate SPM and biopesticides into IPM programs.
- Provide incentives to change the paradigm of pesticide testing from primarily small plot standalone trials to more on-farm larger plot and integrated programs with marketable yield as the ultimate measure of performance and more flexibility in statistical analysis from 0.05 to 1.0 *p*-value.

B. States across the US tailor the principles of SPM such as the California roadmap (https://www.cdpr.ca.gov/docs/sustainable_pest_management_roadmap/) to their state IPM programs.

C. Reduce economic risk for growers transitioning to SPM through payments funded by the USDA as currently done for cover crops and minimum tillage practices.

- Carbon credit payments to reduce carbon footprint/ghg emissions through the use of biologicals.
- Payments to increase on-farm biodiversity.
- Payments to increase soil health using biologicals.
- Payments to incorporate SPM.

## Data Availability

The original contributions presented in the study are included in the article/supplementary material. Further inquiries can be directed to the corresponding author.
